# Primary Soft Tissue Sarcomas of the Extremities: A Nationwide Retrospective Cohort Study of Histology-Specific Outcomes and Prognostic Factors

**DOI:** 10.3390/cancers18152515

**Published:** 2026-08-05

**Authors:** Marko Novak, Andraž Perhavec, Barbka Novak Supe, Olga Blatnik, Marija Skoblar Vidmar, Mojca Unk, Sonja Kramer, Saša Marušič, Manuel Ramanović

**Affiliations:** 1 Division of Surgical Oncology, Institute of Oncology Ljubljana, Zaloška cesta 2, SI-1000 Ljubljana, Slovenia; aperhavec@onko-i.si (A.P.); skramer@onko-i.si (S.K.); 2 Faculty of Medicine, University of Ljubljana, Kongresni trg 12, SI-1000 Ljubljana, Slovenia; mskoblar@onko-i.si (M.S.V.); munk@onko-i.si (M.U.); 3 Division of Anesthesiology and Intensive Care, Institute of Oncology Ljubljana, Zaloška cesta 2, SI-1000 Ljubljana, Slovenia; bsupe@onko-i.si; 4 Department of Pathology, Institute of Oncology Ljubljana, Zaloška cesta 2, SI-1000 Ljubljana, Slovenia; oblatnik@onko-i.si; 5 Department for Radiotherapy, Institute of Oncology Ljubljana, Zaloška cesta 2, SI-1000 Ljubljana, Slovenia; 6 Division of Medical Oncology, Institute of Oncology Ljubljana, Zaloška cesta 2, SI-1000 Ljubljana, Slovenia; 7 Department for Radiology, Institute of Oncology Ljubljana, Zaloška cesta 2, SI-1000 Ljubljana, Slovenia; smarusic@onko-i.si

**Keywords:** extremity, soft tissue sarcoma, histology, surgical margins, radiotherapy, survival, referral center

## Abstract

**Background/Objectives**: The aim of this study was to determine the oncologic outcomes for adult patients with primary extremity soft tissue sarcoma (ESTS) treated at the sarcoma referral center in the Republic of Slovenia. **Methods**: Patients from a prospectively maintained institutional database treated between January 2009 and December 2023 were retrospectively analyzed. The cohort was stratified into a high-risk group (HRG) and a low-risk group (LRG). Survival analyses focused on the HRG. Multivariable Cox models were constructed for local recurrence-free survival (LRFS) and distant metastasis-free survival (DMFS), and predictors of major wound complications were evaluated using multivariable logistic regression. **Results**: Among 315 included patients, 242 (76.8%) were in the HRG. In this group the median age was 61.5 years, 82.6% of tumors were in the lower extremity, and median tumor size was 9.0 cm. The most common histological subtype was undifferentiated pleomorphic sarcoma (28.5%). Clear margins were achieved in 86.8%, major postoperative complications occurred in 19.0%, and 60.7% of patients underwent radiotherapy. Local recurrence developed in 11.2%, regional recurrence in 5.0%, and distant metastasis in 32.6%. The corresponding 5-year overall survival, disease-specific survival, LRFS, and DMFS were 69.8%, 73.7%, 87.9%, and 66.8% in the HRG, respectively. In the LRG, only one local recurrence occurred and the 5-year LRFS was 100.0%. Preoperative radiotherapy showed a borderline association with major wound complications in the HRG (OR 2.70, 95% CI 1.00–7.28, *p* = 0.050). Tumor grade and size remained independent predictors of distant metastases, whereas margin status was not significantly associated with LRFS or DMFS. The primary amputation rate in the whole series was 2.9%. **Conclusions**: Treatment of primary ESTS patients in a specialized national referral cancer center achieved good overall survival (69.8%), a high limb-salvage rate (97.1%), and good local control, affirming that routine primary amputation is rarely needed. Outcomes in high-risk histologies remained driven mainly by distant metastases and showed clear histology-specific differences, whereas low-risk histologies had excellent outcomes with surgery alone. Preoperative radiotherapy was associated with a higher risk of major wound complications.

## Simple Summary

Extremity soft tissue sarcomas (ESTSs) are rare, biologically heterogeneous mesenchymal malignancies that are best managed in specialized sarcoma referral centers. This nationwide retrospective cohort study analyzed 315 adult patients with primary localized ESTS treated at the only sarcoma referral center in the Republic of Slovenia. Patients were stratified into a high-risk group (HRG) and a low-risk group (LRG) based on histological subtype and metastatic potential. In the HRG, the 5-year overall survival was 69.8%, and the 5-year distant metastasis-free survival was 66.8%. High-risk tumors showed good limb preservation and local control but substantial distant failure, with pronounced histology-specific patterns: rhabdomyosarcoma and undifferentiated pleomorphic sarcoma had the highest metastatic rates, whereas myxofibrosarcoma carried the greatest local recurrence risk. Preoperative radiotherapy showed a borderline association with higher wound morbidity, while a microscopically negative margin of at least 1 mm significantly lowered the risk of local recurrence. Low-risk tumors had excellent outcomes with surgery alone, supporting treatment and follow-up de-escalation in this subgroup.

## 1. Introduction

Soft tissue sarcomas (STSs) are rare mesenchymal malignancies accounting for approximately 1% of adult cancers [1,2,3,4]. Their reported estimated annual incidence in Europe is 4–5 cases per 100,000 [1]. Clinical manifestations of STSs vary. They can grow rapidly or slowly, be painful or painless, and be located on the body surface or deep within the body [5]. Most patients present with a painless lump that increases in size over a period of time [6]. Median age at diagnosis is about 60 years [1,2,3,4]. Around 50% of STSs are located in the extremities and limb girdles, with the thigh being the most common anatomical location [5,6]. Extremity and girdle tumors thus form a major clinical subgroup. However, their shared anatomical location should not obscure the substantial biological heterogeneity among histological subtypes, which differ substantially in patterns of local growth, metastatic potential, treatment sensitivity, and survival [1,7,8,9,10,11].

The treatment paradigm for extremity STS (ESTS) has shifted from routine amputation towards limb-sparing surgery supported by radiotherapy (RT), reconstructive surgery, and selected systemic therapy. Prospective randomized evidence and long-term follow-up established limb-sparing surgery combined with RT as a safe standard for local control in most patients, allowing preservation of the affected extremity [12,13,14].

**Figure 1:**
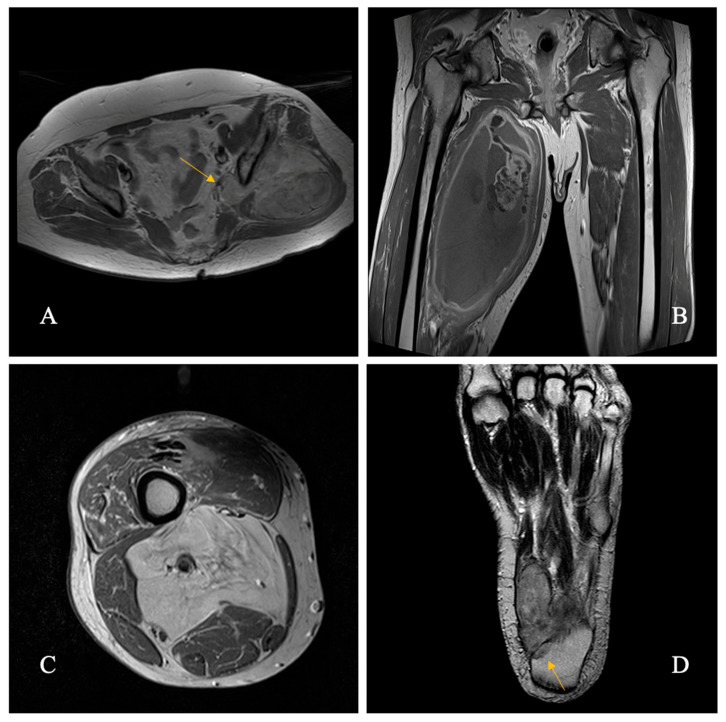
Examples of technically demanding resections (magnetic resonance images): An undifferentiated pleomorphic sarcoma extending through the sciatic notch into the pelvis (orange arrow), removed with a combined anterior and gluteal approach (**A**). An extraskeletal chondrosarcoma with a giant cystic component involving adductor and flexor muscle groups (**B**). An atypical lipomatous tumor encasing the popliteal artery and vein (**C**). A synovial sarcoma invading the medial part of calcaneus (orange arrow), necessitating partial calcaneus resection and a gracilis free flap (**D**). With the exception of case B, clear margins were achieved in all presented cases. Courtesy of the institutional image database.

Multidisciplinary planning is mandatory for patients with ESTS [1,2,15,16]. Resection of ESTS can be technically challenging, requiring coordination between surgical, reconstructive, and RT teams because tumors may be large, deep, or adjacent to critical neurovascular structures (Figure 1). In selected patients with locally advanced or limb-threatening ESTS, where standard resection would cause unacceptable morbidity or require amputation, isolated limb perfusion (ILP) with regional chemotherapy (CT) can be considered [17]. The role of systemic CT in localized ESTS remains selective and debated because randomized trials and meta-analyses have shown variable benefits, with a stronger rationale in selected high-risk or chemo sensitive histological subtypes rather than in unselected STS populations [7,18,19,20].

Soft tissue sarcomas can be locally aggressive with low metastatic potential in lower-risk histologies, whereas aggressive or high-grade histologies are characterized by a higher propensity for local recurrence (LR) and distant metastasis (DM) [1,4,21,22,23]. Long-term outcome in high-risk ESTS patients remains poor. Contemporary prognostic models and large institutional series have shown that survival is predominantly determined by tumor size, grade, depth, histological subtype, age, and surgical margin status. Despite advances in multidisciplinary management, the long-term overall survival (OS) in population at high risk for adverse events remains around 60% [7,9,21,22,23,24,25].

The aim of this study was to determine the oncologic outcomes, treatment modalities, quality of surgery, and prognostic factors in adults with primary localized ESTS treated at the Institute of Oncology Ljubljana Cancer Center (IOLCC), the only sarcoma referral center in the Republic of Slovenia. By excluding patients with primary metastatic disease, recurrent disease, and residual tumors after inadequate surgery at other institution, this cohort focuses specifically on planned primary management and provides a nationwide benchmark for centralized ESTS care.

## 2. Materials and Methods

### 2.1. Study Design and Setting

**Figure 2:**
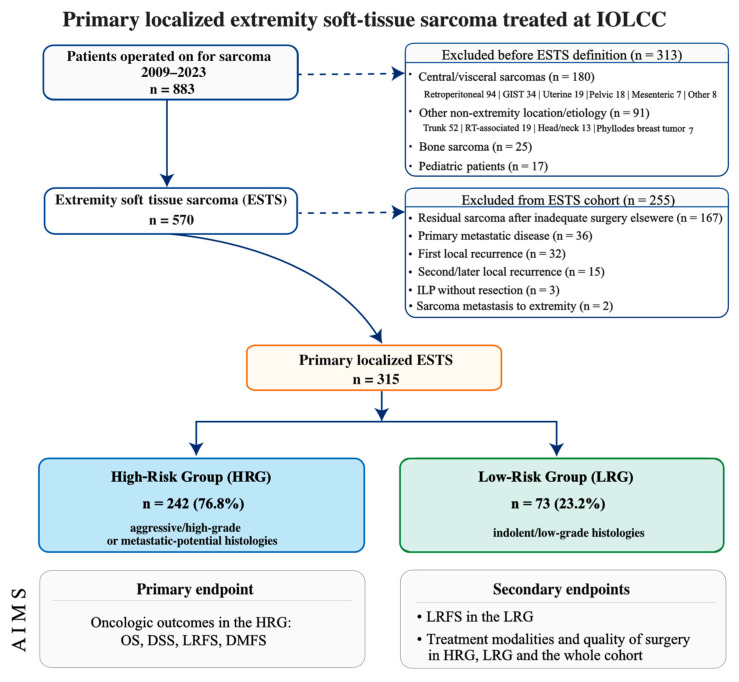
Study flow chart. Selection of patients with primary extremity soft tissue sarcoma included in the study. DMFS, distant metastasis-free survival; DSS, disease-specific survival; ESTS, extremity soft tissue sarcoma; HRG, high-risk group; IOLCC, Institute of Oncology Ljubljana Cancer Center; LRFS, local recurrence-free survival; LRG, low-risk group; OS, overall survival.

We conducted a retrospective cohort study at the IOLCC. All consecutive adult patients recorded in the prospectively maintained institutional sarcoma database who were treated for primary localized ESTS between January 2009 and December 2023 were screened for eligibility. Predefined anatomical and disease-related exclusion criteria were then applied, as summarized in Figure 2. The variables analyzed included patient-related factors (sex, age at diagnosis, and American Society of Anesthesiologists (ASA) classification); pathological factors (primary tumor site, depth, tumor size, tumor grade, and histological subtype), and surgical factors (margin status, duration of surgery, blood loss during surgery, hospital stay, and 90-day complication rate).

A tumor was considered primary if patients had not undergone any surgical procedure except surgical biopsy, and localized if there was no evidence of regional or distant metastases at the referral. Local recurrence was defined as recurrence within or directly adjacent to the previous primary tumor bed or operative field. Regional nodal recurrence was defined as recurrence in the anatomically draining regional lymph node basin without direct continuity with the primary tumor bed. Distant metastasis was defined as metastatic disease outside the primary operative field and regional nodal basin.

For the purposes of the present analysis, histological entities were assigned to a study-specific high-risk group (HRG) or low-risk group (LRG) according to their recognized biological behavior and metastatic potential. The HRG comprised histologies with clinically meaningful metastatic potential and/or established aggressive behavior, whereas the LRG comprised predominantly indolent or low-grade entities with very low metastatic risk. This framework was developed for the present study only and is not an externally validated prognostic model. Solitary fibrous tumors were assigned according to the Demicco risk model. Surgical complications were graded according to the Clavien–Dindo (CD) classification [26]. Major wound complications were analyzed separately within the HRG and were defined using wound-complication details together with major morbidity and/or reoperation, to distinguish wound-specific morbidity from overall postoperative morbidity. To test the robustness of this endpoint, a stricter core wound-complication sensitivity definition was also applied, limited to wound infection or abscess, wound dehiscence, delayed wound healing, flap or skin graft necrosis, or wound-related reoperation. Isolated persistent seroma and isolated postoperative bleeding were excluded from the core sensitivity endpoint.

Histological type was determined according to the 2020 World Health Organization Classification of tumors of soft tissue and bone [4]. Low grade fibromyxoid sarcoma was included in the LRG in spite of having a potential for local relapse and/or distant metastasis after a long period of time, even after decades, and having a potential for disease progression or relapse as a high-grade sarcoma. Solitary fibrous tumor (SFT) cases were classified according to the 4-tiered risk stratification model [27]. Patients with SFT classified as having intermediate risk for metastasis were included in the HRG, and cases classified as low-risk were included in the LRG.

The proximal level of the extremity was defined according to the potential amputation level. Interthoracoscapular (forequarter) amputation is the most proximal on the upper extremity, while external hemipelvectomy (hindquarter amputation) is the most proximal on the lower extremity. Therefore, patients with tumors of the shoulder, axilla, and gluteal or inguinal region were also included in the study. The tumors were characterized as superficial or deep according to the fascia level. All patients underwent surgery and received RT and/or systemic CT in accordance with the recommendations of the institutional sarcoma multidisciplinary tumor board.

For irradiated patients, RT timing, prescribed total dose, dose per fraction, number of fractions, treatment dates, and course completion were retrospectively abstracted from the RT record. The study was approved by our institutional review board and ethical committee (ERIDNPVO-0114/2025).

### 2.2. Exclusion Criteria

Patients with central STS, secondary sarcoma, head, neck or truncal tumor location, phyllodes tumor of the breast, bone sarcoma, residual STS after inadequate initial operation at other institution, and primary metastatic or recurrent disease, as well as pediatric patients with STS, were excluded (Figure 2).

### 2.3. Study Endpoints

The primary endpoint of the study was to analyze the oncologic outcomes by determine the OS, disease-specific survival (DSS), local recurrence-free survival (LRFS) and distant metastasis-free survival (DMFS) of the HRG. OS was defined as time from surgery to death from any cause. For DSS, deaths unrelated to sarcoma were excluded. LRFS and DMFS were defined as time from surgery to local recurrence or distant metastasis, respectively. The secondary endpoints were to determine the LRFS for the LRG, and to analyze the treatment modalities and quality of surgery for both cohorts and for the whole series. Surgical margin status and quantitative margin width were evaluated separately. Margin status was defined as R0 when no tumor was present at the inked resection surface and R1 when a tumor was present. Therefore, this binary variable was included in the multivariable Cox models. Margin width was examined in a separate exploratory Kaplan–Meier analysis using the categories R1, R0 with a closest recorded margin of 1–5 mm, and R0 with a closest recorded margin of >5 mm.

### 2.4. Statistical Methods

Continuous variables are presented as medians (interquartile range, Q1–Q3) or means with range, as appropriate, while categorical variables are presented as counts and percentages. Survival endpoints (OS, DSS, LRFS, and DMFS) were estimated using the Kaplan–Meier method from the date of surgery to the event of interest or censoring. Deaths unrelated to sarcoma were censored for DSS. Median follow-up was estimated using the reverse Kaplan–Meier method. Five-year survival estimates are reported with 95% confidence intervals. Selected subgroup comparisons were assessed using the log-rank test, and regional recurrence (RR) was analyzed descriptively based on the number of events. Multivariable logistic regression was used to identify predictors of major wound complications. Multivariable Cox proportional hazards models were constructed for LRFS and DMFS. Proportional hazards assumption was tested using Schoenfeld residuals [28].

All *p*-values were two-sided, and *p* < 0.05 was considered statistically significant. No adjustment for multiple comparisons was applied because of the exploratory nature of the study. The analysis was performed using R statistical software (version 4.3.2, R Core Team, R Foundation for Statistical Computing, Vienna, Austria).

## 3. Results

### 3.1. Cohort Characteristics

During the 15-year study period, 315 patients with primary localized ESTS were treated at our institution. Of these patients, 242 (76.8%) were included in the HRG cohort, and 73 (23.2%) in the LRG cohort. Rare histologies in the HRG cohort included extraskeletal osteosarcoma (n = 2), small round cell tumor (n = 1), *CIC*-rearranged sarcoma (n = 1), myofibroblastic sarcoma (n = 1) and myoepithelial carcinoma (n = 1). Rare histologies in the LRG included low-grade fibromyxoid sarcoma (n = 3), pleomorphic hyalinizing angiectatic tumor, superficial CD34+ fibroblastic tumor, and atypical myxoid neoplasm, one of each. The median age of the whole cohort was 61.0 years (IQR 49.0–72.0) with a slight male predominance (56.0%). Lower extremity was the most frequent tumor site (80.6%), and 65.4% of lesions were deep to fascia. Median tumor size was 9.0 cm overall, 8.0 cm in the HRG cohort, and 10.5 cm in the LRG cohort. Demographic and clinicopathological data for the cohorts are presented in Table 1.

**Figure 3:**
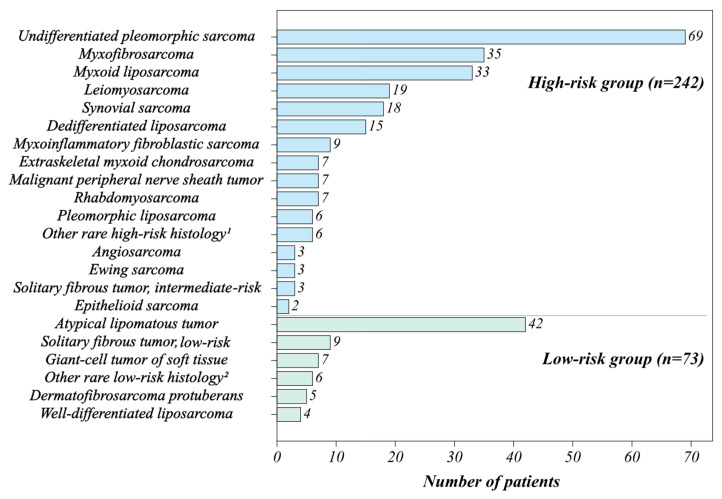
Histology distribution by risk group. ^1^ Rare histologies in the HRG included extraskeletal osteosarcoma, small round cell tumor, CIC-rearranged sarcoma, myofibroblastic sarcoma and myoepithelial carcinoma. ^2^ Rare histologies in the LRG included low-grade fibromyxoid sarcoma, pleomorphic hyalinizing angiectatic tumor, superficial CD34+ fibroblastic tumor and atypical myxoid neoplasm.

The histological spectrum in the HRG cohort was dominated by undifferentiated pleomorphic sarcoma (UPS; 69/242, 28.5%), myxofibrosarcoma (MFS; 35/242, 14.5%), and myxoid liposarcoma (LPS; 33/242, 13.6%), followed by leiomyosarcoma (19/242, 7.9%), synovial sarcoma (18/242, 7.4%), and dedifferentiated LPS (15/242, 6.2%). The LRG cohort was dominated by atypical lipomatous tumor (ALT; 42/73, 57.5%), with smaller contributions from low-risk SFT (9/73, 12.3%), giant-cell tumor of soft tissue (7/73, 9.6%), dermatofibrosarcoma protuberans (DFSP; 5/73, 6.8%), well-differentiated liposarcoma (4/73, 5.5%), and other rare, low-grade entities. A detailed histology distribution is shown in Figure 3.

Treatment modalities, quality of surgery, and oncologic outcomes are presented in separate sections for the HRG cohort, LRG cohort, and for the whole study cohort.

### 3.2. Treatment Modalities and Surgical Quality

#### 3.2.1. High-Risk Group

Radiotherapy was administered to 147 of the 242 HRG patients (60.7%). Of these patients, 44 (29.9% of those irradiated) received preoperative RT and 103 (70.1%) received postoperative RT (Table 2). All preoperative courses were completed as planned, whereas postoperative RT was interrupted in three patients (2.9% of postoperative courses) due to the disease progression: two with distant metastases (synovial sarcoma and malignant peripheral nerve sheath tumor) and one with RR (MFS). Preoperative RT was delivered with a standard regimen of 50 Gy in 25 fractions over five weeks to the gross tumor and a margin. Postoperative RT typically comprised 60–66 Gy in 30–33 fractions to the surgical bed and a margin, with dose tailoring according to margin status, proximity of critical structures, and wound-healing status. During the study period, three-dimensional conformal RT was used predominantly in the early years, with a progressive transition to intensity-modulated RT (IMRT) and volumetric modulated arc therapy (VMAT) for deep or anatomically complex tumors, particularly in the lower extremity. The indication for preoperative versus postoperative RT was determined by the multidisciplinary sarcoma board, taking into account tumor size, depth, anatomical constraints, anticipated wound morbidity, and patient-related factors. Systemic CT was employed in 40 patients (16.5%): 16 (40.0% of these received CT) in the preoperative setting, 19 (47.5%) in the postoperative setting, and five (12.5%)—all with Ewing sarcoma (n = 3) or rhabdomyosarcoma (n = 2), in both settings. Doxorubicin-based regimens were the mainstay of CT, used in 33 of the 40 patients (82.5%). Combined preoperative RT-CT was delivered to 14 patients (9.5% of those irradiated) and postoperative RT-CT to 18 patients (12.2%).

Microscopically negative surgical margins (R0) were achieved in 210 patients (86.8%). A positive margin was defined as the presence of tumor cells at the inked surface. The mean operating time was 192 min (range 20–840 min), the mean estimated blood loss was 151 mL (range 0–4200 mL), and the mean postoperative hospital stay was 6.9 days (range 0–54 days). Flap reconstruction was required in 53 patients (21.9%), most frequently using a gastrocnemius flap (n = 19, 35.8% of all flaps) or a local flap (n = 11, 20.8% of all flaps). Two patients underwent free tissue transfer for a complex foot defect using the latissimus dorsi and gracilis flap. Major artery resection was performed in six patients: superficial femoral in three cases, and ulnar, popliteal and tibial in one case each. Isolated limb perfusion followed by resection was used in seven patients.

Postoperative complications of CD grade 3a or higher occurred in 46 patients (19.0%). The most frequent major complications were persistent seroma (14/46, 30.4%), wound infection or abscess (11/46, 23.9%), wound dehiscence (7/46, 15.2%), flap or graft necrosis (7/46, 15.2%) and postoperative bleeding (4/46, 8.7%). Reoperation was necessary in 19 of these 46 patients (41.3%): 14 underwent abscess drainage or wound debridement with secondary closure by flap (n = 5) or skin graft (n = 3), four were reoperated on for hemorrhage (21.1%), and one required a flap revision. Two patients in the HRG died postoperatively: one from septic complications following hip disarticulation due to massive bleeding from a ruptured femoral artery in a previously irradiated field, and one from pulmonary infection.

#### 3.2.2. Low-Risk Group

In the LRG, surgery constituted the definitive treatment in virtually all cases. Postoperative RT was administered to only two patients (2.7%)—one with an ALT and a positive surgical margin, and one with a large SFT of the upper arm, which was located in a place where future salvage surgery in case of LR would have been anatomically difficult. Clear margins were achieved in 63 patients (86.3%). The mean operating time was 167 min (range 30–645 min), the mean blood loss was 72 mL (range 0–2500 mL), and the mean hospital stay was 4.6 days (range 0–13 days). Flap reconstruction was necessary for nine patients (12.3%), most of which were local flaps (n = 6).

Major complications (CD ≥ 3a) occurred in 11 patients (15.1%), predominantly consisting of persistent seroma (5/11, 45.5%), wound infection or dehiscence (4/11, 36.4%) and postoperative bleeding (2/11, 18.2%). Three of the 11 patients (27.2%) required reoperation: one for wound debridement and primary closure, one for secondary wound coverage with a skin graft, and one for a rupture of the femoral artery, which necessitated vascular repair and coverage with an anterior thigh pedicle flap. There were no postoperative deaths in the LRG.

#### 3.2.3. Whole Cohort

Clear margins were achieved in 273 out of 315 patients (86.7%) across the entire cohort. The mean operating time was 187 min (range 20–840 min), the mean blood loss was 133 mL (range 0–4200 mL), and the mean hospital stay was 6.4 days (range 0–54 days). Primary amputation was necessary for nine patients (2.9%). Detailed indications and the amputation level are summarized alongside the 90-day morbidity profile in Table 3. Major complications (CD ≥ 3a) were recorded in 57 patients (18.1%), and 22 of these (38.6%) required reoperation.

### 3.3. High-Risk Group—Oncologic Outcomes

In the HRG, the median follow-up estimated by the reverse Kaplan–Meier method was 66.0 months. Local recurrence occurred in 27 out of 242 patients (11.2%), regional recurrence in 12 out of 242 (5.0%), and distant metastasis in 79 out of 242 (32.6%). At the last follow-up, 166 patients were alive, 62 had died of disease, and 14 had died of other causes. Consequently, the all-cause mortality rate in the HRG was 31.0%.

Local recurrence occurred in 27 patients (11.2%), regional lymph node recurrence in 12 patients (5.0%) and distant metastasis in 79 patients (32.6%). The median times to these events were 48.6 months (range 3.0–98.2 months), 48.2 months (range 0.7–109.2 months) and 44.0 months (range 0.7–119.7 months), respectively, with corresponding means of 23.5, 18.1 and 20.5 months. Examining the pattern, isolated LR was seen in 4.5% of patients (11/242), while isolated RR occurred in only two cases. Distant metastasis alone—without concurrent or antecedent LR or RR—developed in 24.0% of patients (58/242). The remaining patients presented as follows: seven had concomitant LR and DM, four developed LR followed by DM, four had concomitant RR and DM, two had DM followed by RR, two had DM followed by LR, one had RR followed by DM, and one patient presented with concomitant LR and DM, later developing RR.

**Figure 4:**
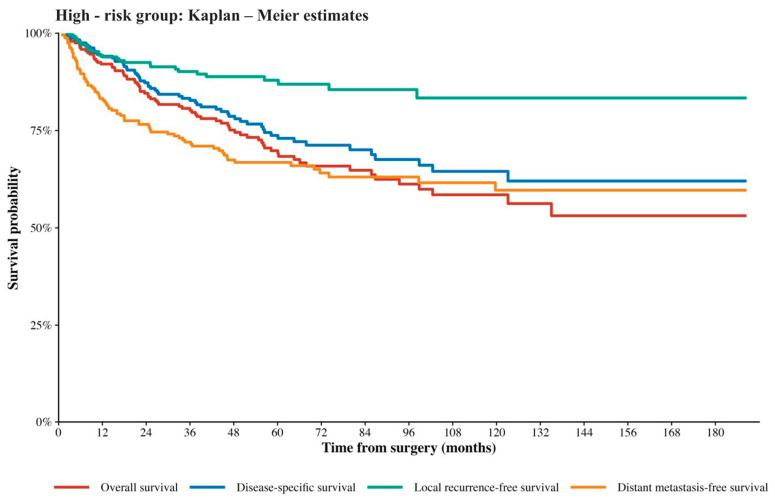
High-risk group Kaplan–Meier estimates for overall survival, disease-specific survival, local recurrence-free survival, and distant metastasis-free survival.

Five-year Kaplan–Meier estimates were 69.8% for OS, 73.7% for DSS, 87.9% for LRFS and 66.8% for DMFS (Figure 4).

As detailed in Table 4, histology-specific outcomes revealed that epithelioid sarcoma, extraskeletal myxoid chondrosarcoma, MFS and myxoinflammatory fibroblastic sarcoma had the highest LR rates, whereas rhabdomyosarcoma, malignant peripheral nerve sheath tumor and UPS were the most frequently metastasizing subtypes.

### 3.4. Low-Risk Group—Oncologic Outcomes

The LRG had a median follow-up time of 60.0 months. Only one patient developed LR, which occurred 106.1 months after surgery. There was no RR or DM among low-risk patients. Five-year LRFS was 100.0%.

### 3.5. Multivariable Cox Models for LRFS and DMFS

**Figure 5:**
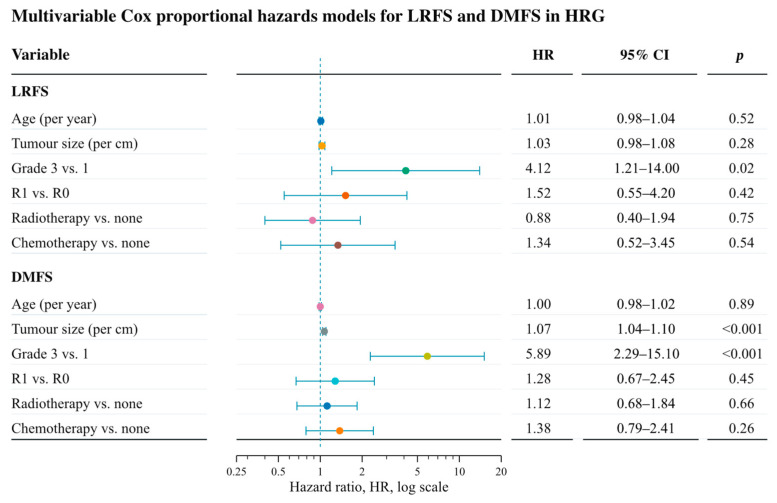
Multivariable Cox proportional hazards models for LRFS and DMFS in the high-risk group. Points represent adjusted hazard ratios and horizontal lines represent 95% confidence intervals. Tumor size and grade 3 disease were significantly associated with inferior DMFS, while grade 3 disease was also associated with inferior LRFS. CI, confidence interval; DMFS, distant metastasis-free survival; HR, hazard ratio; HRG, high-risk group; LRFS, local recurrence-free survival.

For LRFS, high tumor grade (Grade 3 vs. 1: HR 4.12, 95% CI 1.21–14.0, *p* = 0.02) was the only independent predictor. For DMFS, both tumor size (per cm: HR 1.07, 95% CI 1.04–1.10, *p* < 0.001) and grade (Grade 3 vs. 1: HR 5.89, 95% CI 2.29–15.1, *p* < 0.001) were strong independent predictors. Surgical margin status (R1 vs. R0) was not independently associated with either LRFS (HR 1.52, *p* = 0.42) or DMFS (HR 1.28, *p* = 0.45), and neither RT or CT was an independent predictor in the models (Figure 5).

### 3.6. Predictors of Major Wound Complications

Major wound complications were analyzed separately in the HRG using wound-complication details together with major morbidity and/or wound-related reoperation. This endpoint was defined to distinguish between wound-specific morbidity and major postoperative morbidity. In the HRG, major wound complications occurred in 42 out of 242 patients (17.4%). According to RT timing, major wound complications occurred in 15 out of 95 patients (15.8%) who were treated without RT, 10 out of 44 patients (22.7%) who were treated with preoperative RT and 16 out of 103 patients (15.5%) who were treated with postoperative RT. Local wound complications were more frequent after preoperative RT than after postoperative RT (40.5% vs. 23.3%, *p* = 0.044), whereas the difference in the primary major wound-complication endpoint did not reach statistical significance (23.8% vs. 15.5%, *p* = 0.243).

**Figure 6:**
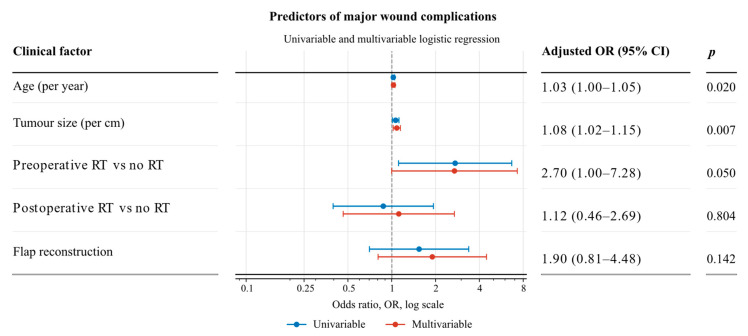
Predictors of major wound complications in the high-risk group. Forest plot showing univariable and multivariable logistic regression estimates. Points represent odds ratios and horizontal lines represent 95% confidence intervals. Blue points represent univariable estimates and red points represent multivariable estimates. Adjusted odds ratios, 95% confidence intervals, and *p*-values shown in the right-hand columns refer to the multivariable model. Major wound complications were defined using wound-complication details together with major morbidity and/or reoperation. OR, odds ratio; CI, confidence interval; RT, radiotherapy.

Multivariable logistic regression analysis, which included age, tumor size, tumor grade, RT timing, and flap reconstruction, revealed that older age and larger tumor size were associated with higher odds for major wound complications. As shown in Figure 6, each additional year of age was associated with an increased risk (OR 1.03, 95% CI 1.00–1.05, *p* = 0.020), as was each additional centimeter of tumor size (OR 1.08, 95% CI 1.02–1.15, *p* = 0.007).

The timing of RT showed different associations according to the treatment sequence. Preoperative RT showed a borderline association with increased major wound-complication risk compared with no RT (OR 2.70, 95% CI 1.00–7.28, *p* = 0.050), whereas postoperative RT was not associated with an increased risk (OR 1.12, 95% CI 0.46–2.69, *p* = 0.804). Flap reconstruction showed a non-significant trend towards higher wound morbidity (OR 1.90, 95% CI 0.81–4.48, *p* = 0.142).

### 3.7. Margin Status and Margin-Width Analysis

Margin status and margin width were examined as distinct variables. As reported in the multivariable Cox model, binary margin status (R1 vs. R0) was not independently associated with LRFS after adjustment for the other included clinicopathological and treatment variables (HR 1.52, 95% CI 0.55–4.20, *p* = 0.42).

**Figure 7:**
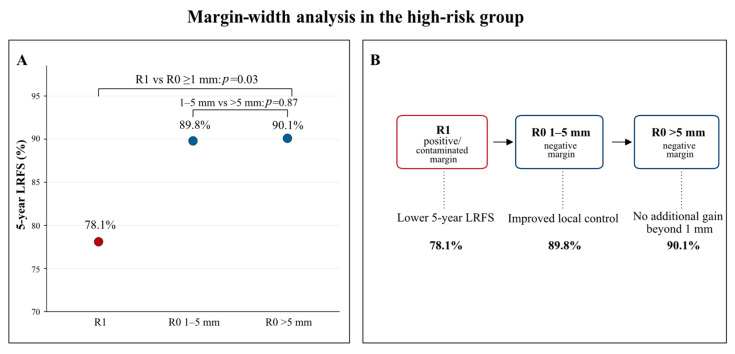
Exploratory margin-status and margin-width analysis of local recurrence-free survival in the high-risk group. Five-year local recurrence-free survival was lower in patients with positive or contaminated margins than in patients with microscopically negative margins of at least 1 mm (**A**). Among patients with R0 resection, increasing margin width beyond 1 mm did not provide additional improvement in 5-year local recurrence-free survival (**B**). LRFS, local recurrence-free survival; R0, microscopically negative margin; R1, positive or contaminated margin.

In the separate exploratory, unadjusted margin-width analysis, 5-year LRFS in HRG cohort was 78.1% for patients with R1 resections, 89.8% for patients with R0 margins of 1–5 mm, and 90.1% for patients with R0 margins of >5 mm. Patients with R1 margins had lower LRFS than patients with R0 margins of at least 1 mm (*p* = 0.03). Among patients with R0 resections, no difference was observed between margins of 1–5 mm and >5 mm (*p* = 0.87), shown in Figure 7.

### 3.8. Regional Nodal Recurrences

Twelve patients developed RR, with UPS accounting for five of these cases and the remaining seven distributed across synovial sarcoma, rhabdomyosarcoma, Ewing sarcoma, epithelioid sarcoma, extraskeletal myxoid chondrosarcoma, MFS and leiomyosarcoma. The median time to nodal recurrence was 48.2 months (range 0.7–109.2 months).

## 4. Discussion

The Republic of Slovenia has a population of 2.1 million. Established in 1938, the Institute of Oncology Ljubljana was certified by the Organisation of European Cancer Institutes (OECI) as a Cancer Centre in 2022. As the country’s only sarcoma referral center, it has a multidisciplinary sarcoma team of 24 specialists. The centralized management and treatment of sarcoma patients in a single institution offers the unique opportunity to evaluate these data as an observational nationwide cohort study. This study involved the treatment of 315 consecutive patients with primary localized ESTS over a 15-year period, providing a comprehensive national benchmark for ESTS care.

Patients with large and deep soft tissue tumors are more likely to be referred to specialized sarcoma center, whereas patients with superficial and small soft tissue tumors are more likely to undergo unplanned excision by a non-sarcoma surgeon. Residual STSs after unplanned excision are potentially associated with a worse prognosis for LR and, in most cases, reoperation is indicated. Following a literature review, Nakamura et al. reported that patients who underwent reoperation after unplanned excision of STS did not experience higher mortality or local failure compared to those who underwent planned resection [29]. However, reoperations can be more extensive than primary resections and often result in more complex reconstructive procedures, with a higher risk of postoperative complications [29]. In order to provide a clean benchmark for planned primary management, this study deliberately excluded patients with residual ESTS after unplanned excision at other institutions, as well as those with recurrent or primary metastatic disease, meaning the cohort focused on patients who were managed with upfront, multidisciplinary-team-planned therapy within the national referral center. To our knowledge, this is the first study to stratify the STS study population into an HRG and LRG.

### 4.1. Overall and Disease-Specific Survival

In the HRG, the 5-year OS of 69.8% and DSS of 73.7% are comparable to outcomes reported from large sarcoma-center series. However, direct comparisons are limited by differences in inclusion criteria, treatment era, anatomical scope, and histology-based comparisons, as well as the use of RT. In the MD Anderson series by Zagars et al., 1225 patients with localized STS who were treated with conservative surgery and RT achieved a 5-year DSS of 73%, with 5-year local control and DMFS of 83% and 71%, respectively [30]. However, that study included a broader population of patients with localized STS, was not restricted to primary extremity tumors, and all patients received RT. Therefore, it should be interpreted as a broad historical benchmark rather than a direct comparison for high-grade ESTS. A more anatomically comparable series of 1041 adult patients with localized ESTS reported a 5-year survival rate of 76%. Tumor size, grade and depth, recurrent presentation, histology, margin status, and lower-extremity site were identified as adverse prognostic factors [31]. The Sarculator nomogram, a landmark tool, developed from 1452 patients with primary localized ESTS and externally validated in independent cohorts, predicts 5-year OS ranging from 50% to 90% depending on tumor size, grade, depth, and histology [27]. Our findings fall within this expected range. The 5-year OS of 69.8% in our HRG is also similar to the 68% reported in a 2024 systematic review of patients receiving RT for ESTS [25]. Notably, DSS was only 4% higher than OS (73.7% vs. 69.8%), indicating that competing non-sarcoma mortality is relatively low in this patient population. This is consistent with a median age of 61.5 years and a low proportion of ASA ≥3 (24.8%) [29]. In comparison to historical series, such as 2006 cohort of the French Sarcoma Group with a 5-year OS of 59%, the relatively favorable OS of our cohort reflects improvements in multidisciplinary management, including better patient selection for RT, refined surgical techniques, and potentially greater efficacy of systemic therapy for chemosensitive histologies [6,20,21,23].

### 4.2. Histology-Specific Outcomes

The histological distribution of HRG tumors—dominated by UPS (28.5%), MFS (14.5%), and myxoid LPS (13.6%)—is consistent with the epidemiology of high-grade ESTS [32]. Histology-specific analyses revealed clinically relevant differences in recurrence patterns that align with the recent literature. In a large comparative series, MFS demonstrated a significantly higher LR rate (34.4% vs. 20.9% for UPS), whereas UPS carried a substantially higher risk of DM (44.2% vs. 12.2% for MFS) and tumor-related death (32.6% vs. 9.5%) [32]. These distinct patterns of failure justify histology-tailored surveillance strategies: MFS requires closer local monitoring, while UPS necessitates heightened vigilance for distant metastatic spread [14].

Emerging data suggest that there are divergent immunogenic features between UPS and MFS. Notably, preoperative RT appears to induce cytotoxic T-cell infiltration and depletion of myeloid cells in UPS, whereas these effects are not observed in MFS. These findings emphasize significant differences in the immunobiology of UPS and MFS, with implications for therapy and prognosis [33]. Such differences may partly explain histology-specific responses to therapy and have implications for future immunotherapy combinations in localized disease.

The selective use of systemic chemotherapy in our cohort (16.5% of HRG patients) aligns with recent real-world evidence from the DEEPSARC nationwide French study, which demonstrated that adjuvant chemotherapy in unselected localized STS populations does not confer a uniform survival benefit, reinforcing the need for histology-guided patient selection [34]. The companion economic evaluation by Perrier et al. further demonstrated that the cost-effectiveness of adjuvant chemotherapy varies substantially by histology and risk group [35]. These economic considerations, while beyond the scope of our clinical analysis, underscore the importance of personalized, histology-guided treatment decisions in sarcoma care. In our cohort, CT was predominantly employed in chemosensitive histologies, including Ewing sarcoma (100%), rhabdomyosarcoma (57.1%), and synovial sarcoma (33.3%), reflecting contemporary practice patterns and the recognition that CT benefit varies substantially by histological subtype.

### 4.3. Limb Salvage and Local Control

The primary amputation rate of 2.9% (9/315) is remarkably low and compares favorably with rates of 3.6–8% reported in major contemporary series from other referral centers [36]. This achievement reflects the effectiveness of limb-sparing surgery, facilitated by advanced reconstructive techniques—including flap reconstruction in 21.9% of HRG patients—and the selective application of ILP in seven patients [17]. Reconstructive surgery should not be regarded solely as a means of closing the resulting defect; when incorporated into preoperative planning, it may allow a more oncologically appropriate resection while providing reliable coverage of exposed neurovascular, osseous, or prosthetic structures.

This interpretation is consistent with the experience of Samà et al., in which planned collaboration between oncologic and plastic-reconstructive surgeons permitted wide resection and flap coverage in anatomically complex extremity or parietal-trunk STS. Among 89 patients requiring flap reconstruction, R0 margins were achieved in 92.1%, despite a population in which an amputation or incomplete resection had otherwise been anticipated [37]. Contemporary ASO guidance similarly emphasizes multidisciplinary planning, negative-margin surgery where safely feasible, preservation of limb function, and integration of reconstructive expertise when the anticipated resection would result in a complex defect [16].

The R0 resection rates of 86.8% in the HRG and 86.7% overall are excellent, falling within the range reported by high-volume sarcoma centers (85–90%) [38,39]. The 5-year LRFS of 87.9% in the HRG demonstrates effective local control. However, isolated LR without concomitant distant spread occurred in only 4.5% of patients, whereas 24% developed distant metastases alone, which underscores the fact that distant dissemination is the dominant cause of treatment failure in high-risk disease [4,5].

### 4.4. Regional Nodal Recurrence

Regional nodal recurrence occurred in 12 HRG patients (5.0%), with UPS accounting for five cases and the remaining events clustering in histologies known to have nodal tropism, including rhabdomyosarcoma, epithelioid sarcoma, and certain tumors associated with specific genetic alterations. Population-based and large institutional series have reported lymph node metastases in approximately 2–5% of adult patients with STS. Large size (>10 cm), high grade, and specific histological subtypes (including clear cell sarcoma and epithelioid sarcoma) act as significant risk factors for regional metastasis at presentation [40,41]. The prognostic impact of isolated regional metastasis differs among subtypes [40]. The observation that some patients with isolated nodal disease can achieve long-term survival after therapeutic lymphadenectomy highlights the fact that nodal involvement is not always fatal and should be treated aggressively in selected cases. Overall, our data support a histology-guided approach to nodal staging and surveillance, using targeted imaging and potentially sentinel node procedures in high-risk histologies, rather than routine nodal staging for all ESTS [42,43,44,45].

### 4.5. Radiotherapy and Wound Complications

Radiotherapy was administered to 60.7% of HRG patients, representing an important component of multimodal limb-sparing treatment. In contemporary practice, the decision to add radiotherapy and the choice of sequencing should be made within a specialist sarcoma multidisciplinary team, balancing oncologic benefit against functional and wound-healing considerations [46,47].

The Canadian randomized trial comparing preoperative with postoperative radiotherapy is best interpreted as demonstrating different toxicity profiles rather than a universally mandatory sequence [48]. Preoperative radiotherapy was associated with a significantly higher rate of acute wound complications, whereas later analysis of the same randomized cohort showed a tendency toward greater late fibrosis, edema, and joint stiffness after postoperative radiotherapy, with these late effects adversely affecting function [49].

The role of RT in ESTS is well established in selected patients, particularly when the risk of LR is clinically relevant or when close margins are anticipated [1,2,13,14,15,16].

Moderate hypofractionation schedules may offer a balanced approach by maintaining oncologic outcomes while improving toxicity and wound-complication profile. A regimen of 14–15 fractions of 3 Gy resulted in a 33% wound-complication rate and a 2-year LR rate of 7.6%, with no increase in postoperative wound complications and favorable local failure rates [50].

In the HRG cohort, older age and larger tumor size were associated with major wound complications, while preoperative RT showed a borderline association with higher wound morbidity compared with no RT.

Modern ESTS series and systematic reviews further support the hypothesis that wound-complication rates vary substantially across cohorts and are influenced by lower-extremity/proximal location, tumor size, RT timing, and local treatment complexity [51,52,53].

The predominance of postoperative radiotherapy in our cohort should therefore be understood as a real-world finding from a long study period (2009–2023), not as evidence against the modern preference for preoperative treatment in selected candidates. Likely contributors include referral patterns in which surgery was already being prioritized, concern about wound complications in larger or lower-extremity tumors, the anticipated need for complex closure or flap reconstruction, and gradual evolution of institutional sequencing practice during the study period [51,54]. Recent radiotherapy-specific guidance favors preoperative RT when RT is indicated, but still emphasizes multidisciplinary selection and modern conformal planning, so our distribution should be interpreted as era-dependent real-world practice rather than deviation from a universal standard [47].

### 4.6. Margin Status, Margin Width, and Local Recurrence

Surgical margin status and quantitative margin width represent related but distinct concepts. Margin status distinguishes an R1 resection, in which tumor reaches the inked surface, from an R0 resection, in which the inked surface is tumor-free. Margin width describes the measured distance between the tumor and the closest negative resection surface. The relationship between surgical margin status and LR remains a topic of intense discussion [7,8]. In our multivariable Cox analysis, margin status was not independently associated with LRFS or DMFS when grade and size were included. However, when HRG patients were stratified by margin width, those with positive margins had a significantly lower 5-year LRFS (78.1%) than those with clear margins of at least 1 mm (89–90%, *p* = 0.03). Margin width beyond 1 mm did not provide any additional benefit for patients with negative margins.

Recent large-scale studies provide an important context for these findings. A 2025 study of 203 patients with primary high-grade STS reported a 5-year LRFS of 11.5% for positive margins, 58% for margins 0–1 mm, 76% for margins >1–5 mm, and 93% for margins >5 mm. The study concluded that, to minimize the risk of LR, a resection margin of at least 5 mm should be attained. When postoperative RT is applied, the likelihood of LR decreases even further. In scenarios where preserving critical structures is essential, a resection margin of less than 5 mm may be acceptable [38]. Another analysis from 2025 of 185 patients found that achieving a negative margin of more than 1 mm was associated with a lower risk of LR (HR 0.41), whereas positive surgical margins had a detrimental impact on OS (HR 3.58) [39].

Taken together, our findings and the available literature support achieving a microscopically negative margin whenever feasible. However, neither the present study nor the existing heterogeneous retrospective evidence establishes a single numerical margin width that is appropriate for all ESTS. The required extent of resection should be individualized according to tumor biology, anatomical barriers, anticipated functional morbidity, and the use of RT. When preserving critical structures is essential and postoperative RT is planned, margins of less than 5 mm may be acceptable [15,38,39]. Accordingly, the operative objective should be an R0 resection while preserving limb function and avoiding disproportionate morbidity. Consistent with contemporary ASO guidance, the adequacy of a close negative margin should be interpreted in relation to histological subtype, anatomical barriers, proximity to critical structures, anticipated functional consequences of a wider resection, and the planned use of RT [16].

### 4.7. Low-Risk Group Management

The low-risk group (23.2% of all patients) achieved excellent outcomes with surgery alone in virtually all cases, with a 5-year LRFS rate of 100%. However, 14% of patients in this cohort (10/73) had a positive surgical margin and only one received postoperative RT, reflecting the low biological potential and tendency for local recurrence. This supports the practice of reserving RT for selected indications, such as positive margins in anatomically critical locations where salvage surgery in case of LR would be challenging [16]. Low-risk histologies nevertheless require individualized long-term surveillance, particularly SFT and low-grade fibromyxoid sarcoma, because late recurrences can occur. A systematic review of SFT outcomes reported that 10–30% of patients experience recurrent disease after surgical resection. High mitotic rate, Ki67 index and presence of necrosis were identified as the most significant risk factors for recurrence. The Demicco risk stratification system remains the most validated and widely used, though G-score models may offer superior discrimination with longer follow-up [55]. For ALT, LR rates of approximately 16% have been reported, with a mean time to recurrence of 48.4 months. This underscores the necessity of long-term surveillance [56]. Our results also show that routine chest imaging during long-term surveillance in the LRG cohort, especially in ALT histology, may be omitted since there was no reported DM [57].

### 4.8. Strengths and Limitations

This study has several strengths. Firstly, it captures all adults with primary localized ESTS who were treated within a clearly defined national population and managed within a single OECI-accredited cancer center with a dedicated multidisciplinary sarcoma team, thereby minimizing referral and treatment heterogeneity. Secondly, the histology-based stratification of patients into the HRG and LRG, combined with multivariable modeling of LRFS, DMFS, and major wound complications, provides clinically granular information that goes beyond aggregated ESTS cohorts. Thirdly, the follow-up period is long enough to characterize medium-term outcomes in the HRG and to confirm the favorable course of most low-risk histologies.

Limitations include the retrospective design and the absence of randomization in the choice and timing of RT and CT, which introduces residual confounding factors. The 15-year accrual period spans advance in imaging, reconstruction, RT techniques, and systemic therapies, meaning that outcomes reflect evolving practice rather than a single contemporary protocol. Some histological subgroups remain small despite nationwide accrual, which limits the precision of subtype-specific estimates and the stability of multivariable models. Margins are assessed using pathological sampling, which might underestimate three-dimensional infiltrative growth, particularly in MFS and UPS [38,39]. Late RT effects, including fibrosis, edema, joint stiffness, neuropathy, fracture, and long-term functional impairment, were not systematically recorded in the study database. Consequently, the present study cannot compare the long-term toxicity profiles of preoperative and postoperative RT. Finally, follow-up may still be insufficient to capture very late recurrences in entities such as myxoid LPS and low-grade fibromyxoid sarcoma.

## 5. Conclusions

Treatment of primary ESTS patients at the Institute of Oncology Ljubljana achieved a high OS rate of 69.8%, an excellent primary limb-salvage rate of 97.1%, and good local control (5-year LRFS of 87.9% in HRG, and 100% in LRG). In patients with high-risk disease, outcomes were mainly driven by DM (32.6% 5-year metastatic rate). Rhabdomyosarcoma (71.4%) and UPS (44.9%) demonstrated the highest metastatic propensities, whereas MFS (22.9%) and extraskeletal chondrosarcoma (28.6%) were associated with the greatest LR risk. Tumor grade and size emerged as the principal independent predictors of DM. Achieving a negative margin of more than 1 mm was associated with a reduced risk of LR, with no incremental benefit beyond 1 mm. Preoperative RT showed borderline significance in increasing early wound-healing complications (OR 2.70, 95% CI 1.00–7.28, *p* = 0.050), though long-term toxicity data were not available for comparison. Local control was excellent regardless of RT timing (5-year LRFS 87.9% in HRG), supporting the use of an approach based on individual patient and tumor characteristics, as recommended by contemporary guidelines. The choice between preoperative and postoperative RT should consider the trade-off between early wound complications and potential long-term toxicity benefits. Patients with low-risk histologies had excellent outcomes with surgery alone.

Overall, this nationwide cohort study provides a comprehensive benchmark for ESTS care in a centralized healthcare system. The excellent outcomes underscore the value of specialized, multidisciplinary sarcoma management. Future efforts should focus on refining risk stratification, minimizing treatment-related morbidity, and developing effective systemic therapies to improve DMFS in high-risk histological subtypes.

## Figures and Tables

**Table 1:** Demographics and clinicopathologic details of the high-risk cohort, low-risk cohort and whole cohort.

Characteristic	HRG n = 242 ^1^	LRG n = 73 ^1^	Overall n = 315 ^1^
*Age, years*	61.0 (19, 92)	61.0 (23, 80)	61.0 (19, 92)
*Sex*			
Female	109 (45.0)	30 (41.1)	139 (44.1)
Male	133 (55.0)	43 (58.9)	176 (55.9)
*ASA*			
1	45 (18.6)	18 (24.7)	63 (20.0)
2	127 (52.5)	41 (56.2)	168 (53.3)
3	60 (24.8)	12 (16.4)	72 (22.9)
4	10 (4.1)	2 (2.7)	12 (3.8)
*Site*			
Upper extremity	42 (17.4)	19 (26.0)	61 (19.4)
Lower extremity	200 (82.6)	54 (74.0)	254 (80.6)
Thigh	112 (46.3)	36 (49.3)	148 (47.0)
*Tumor depth*			
Superficial	87 (36.0)	22 (30.1)	109 (34.6)
Deep	155 (64.0)	51 (69.9)	206 (65.4)
*Tumor size* (cm)	9.0 (1.0, 40.0)	8.9 (1.0, 30.0)	9.0 (1.0, 40.0)
*Tumor grade (FNCLCC)*			
1	54 (22.3)	54 (74.0)	108 (34.3)
2	45 (18.6)	0	45 (14.3)
3	143 (59.1)	0	143 (45.4)
NA	0	19 (26.0)	19 (6.0)
*AJCC Stage (8th Edition)*			
I	54 (22.3)	73 (100.0)	127 (40.3)
II	45 (18.6)	0	45 (14.3)
III	143 (59.1)	0	143 (45.4)
IV	0	0	0

Abbreviations: ^1^ Median (Q1, Q3); n (%); HRG, high-risk group; LRG, low-risk group; ASA, American Society of Anesthesiologists classification; FNCLCC, Fédération Nationale des Centers de Lutte Contre le Cancer; AJCC, American Joint Committee on Cancer; NA, not applicable.

**Table 2:** Tumor size, surgical quality and treatment modalities according to histology.

	n	Size, (cm)	Tumor Size, Median (IQR)	R0 (%)	ILP (%)	Flap (%)	RT (%)	CT (%)
HIGH-RISK GROUP								
*UPS*	69	11.5	9 (6, 15)	60 (87.0)	0	12 (17.4)	51 (73.9)	11 (16.2)
*Myxofibrosarcoma*	35	7.7	7 (4.8, 10)	31 (88.6)	0	6 (17.1)	18 (51.4)	1 (2.9)
*Myxoid LPS*	33	13.6	13 (8.7, 18)	32 (97.0)	2	7 (21.2)	16 (48.5)	10 (30.3)
*Leiomyosarcoma*	19	5.3	3.5 (3, 6)	18 (94.7)	2	7 (36.8)	9 (47.4)	0
*Synovial sarcoma*	18	5.9	4.2 (3.1, 7.8)	16 (88.9)	1	5 (27.8)	9 (50.0)	6 (33.3)
*Dedifferentiated LPS*	15	13.1	11 (8.5, 19)	11 (73.3)	0	1 (6.7)	9 (60.0)	2 (13.3)
*MFBS*	9	4.7	5 (3, 5.5)	3 (33.3)	1	5 (55.6)	6 (66.7)	0
*Extraskeletal myxoid HS*	7	12.9	10 (8.5, 14.5)	5 (71.4)	0	2 (28.6)	6 (85.7)	0
*MPNST*	7	11.7	10.5 (7.5, 17)	6 (85.7)	0	1 (14.3)	4 (57.1)	0
*Rhabdomyosarcoma*	7	12.1	10 (6.8, 17)	6 (85.7)	0	0 (0.0)	6 (85.7)	4 (57.1)
*Pleomorphic LPS*	6	12.1	10 (6.8, 17)	5 (83.3)	0	2 (33.3)	5 (83.3)	1 (12.5)
*Angiosarcoma* *	3	3.3	3 (2, 4.5)	3 (100.0)	0	1 (33.3)	2 (66.6)	1 (33.3)
*Ewing sarcoma*	3	5.0	7 (3.5, 7.5)	3 (100.0)	0	0 (0.0)	1 (33.3)	3 (100.0)
*SFT (IR)*	3	8.7	7 (7, 9.5)	3 (100.0)	0	0 (0.0)	2 (66.7)	0
*Epithelioid sarcoma*	2	6.3	6.2 (5.4, 7.1)	2 (100.0)	1	0 (0.0)	1 (50.0)	0
*Rare histology (HR)*	6	7.4	6.95 (4.0, 10.6)	6 (100.0)	0	3 (50.0)	2 (33.3)	1 (16.7)
LOW-RISK GROUP								
*ALT*	42	18.3	18.2 (15, 24.2)	36 (85.7)	0	1 (2.4)	1 (2.4)	0
*SFT (LR)*	9	4.7	4.5 (2.8, 5.3)	9 (100.0)	0	1 (11.1)	1 (11.1)	0
*Giant-cell tumor*	7	2.7	2.3 (1.5, 3.3)	4 (57.1)	0	1 (14.3)	0	0
*DFSP*	5	5.9	6.5 (5, 8)	5 (100.0)	0	4 (80.0)	0	0
*WD-LPS*	4	15.5	16 (10.8, 20.8)	4 (100.0)	0	0 (0.0)	0	0
*Rare histology (LR)*	6	5.0	4.75 (3, 8.5)	5 (83.3)	0	2 (33.3)	0	0

Abbreviations: UPS, undifferentiated pleomorphic sarcoma; LPS, liposarcoma; MFBS, myxoinflammatory fibroblastic sarcoma; HS, chondrosarcoma; MPNST, malignant peripheral nerve sheath tumor; SFT, solitary fibrous tumor; ALT, atypical lipomatous tumor; DFSP, dermatofibrosarcoma protuberans; WD-LPS, well-differentiated liposarcoma; (IR), intermediate-risk; (HR), high-risk; (LR), low-risk; IQR, interquartile range; ILP, isolated limb perfusion; RT, radiotherapy; CT, chemotherapy. * Size of angiosarcoma according to the final histological report after chemotherapy and resection and not according to clinical or radiologic findings before treatment.

**Table 3:** Morbidity in 90-day postoperative period and reasons for primary amputation in patients from the whole series.

Patient	%	Complication		
19/315	6.0	Chronic seroma (>3 months)		
12/315	3.8	Surgical site infection, abscess		
10/315	3.2	Dehiscence of wound		
6/315	1.9	Postoperative bleeding		
7/62	11.3	Flap/skin graft necrosis		
Case, (ASA)	Sex, age	**Reason for amputation**	**Amputation level**	**Histology**
1 (2)	F, 64	Metatarsal destruction	Below knee	Synovial sarcoma
2 (3)	F, 72	Femur fracture at tumor level caused by fall	Above knee	Myxofibrosarcoma
3 (2)	F, 46	Destruction of phalanx	Finger ray, 2nd toe	Giant-cell tumor
4 (1)	M, 27	Metacarpal infiltration	4th–5th finger with both palms	Epithelioid sarcoma
5 (4)	F, 62	Diabetic, blind, immobile	Above knee	Myxoid liposarcoma
6 (3)	F, 73	Extensive disease, polymorbid	Above knee	Angiosarcoma
7 (3)	F, 84	Dementia, polymorbid	Below knee	UPS
8 (2)	F, 78	Extensive disease	Finger ray, 5th finger	Giant-cell tumor
9 (4)	M, 54	Extensive disease, tetraplegic after car accident	Hip disarticulation	UPS

Abbreviations: ASA, American Society of Anesthesiologists classification; F, female; M, male; UPS, undifferentiated pleomorphic sarcoma.

**Table 4:** Oncologic outcomes according to histology.

	n	LR (%)	RR (%)	DR (%)	ALIVE n	DOD n (*)	AWD n
HIGH-RISK GROUP							
*UPS*	69	9 (13.0)	5 (7.2)	31 (44.9)	39	26 (1)	3
*Myxofibrosarcoma*	35	8 (22.9)	1 (2.9)	6 (17.1)	26	5 (3)	1
*Myxoid LPS*	33	0	0	8 (24.2)	26	6	1
*Leiomyosarcoma*	19	0	1 (5.3)	5 (26.3)	13	3 (1)	2
*Synovial sarcoma*	18	1 (5.6)	1 (5.6)	6 (33.3)	12	6	0
*Dedifferentiated LPS*	15	2 (13.3)	0	5 (33.3)	10	2 (1)	2
*MFBS*	9	2 (22.2)	0	1 (11.1)	8	1	0
*Extraskeletal myxoid HS*	7	2 (28.6)	1 (14.3)	2 (28.6)	5	2	0
*MPNST*	7	1 (14.3)	0	4 (57.1)	4	3	0
*Rhabdomyosarcoma*	7	0	1 (14.3)	5 (71.4)	2	4	1
*Pleomorphic LPS*	6	1 (16.7)	0	2 (33.3)	5	1	0
*Angiosarcoma*	3	0	0	0	2	0 (1)	0
*Ewing sarcoma*	3	0	1 (33.3)	1 (33.3)	2	1	0
*SFT (IR)*	3	0	0	0	3	0	0
*Epithelioid sarcoma*	2	1 (50.0)	1 (50.0)	1 (50.0)	1	1	0
*Rare histology (HR)*	6	0	0	2 (33.3)	4	2	0
LOW-RISK GROUP							
*ALT*	42	1 (2.4)	0	0	42	0	0
*SFT (LR)*	9	0	0	0	9	0	0
*Giant-cell tumor*	7	0	0	0	6	0 (1)	0
*DFSP*	5	0	0	0	5	0	0
*WD-LPS*	4	0	0	0	4	0	0
*Rare histology (LR)*	6	0	0	0	6	0	0

Abbreviations: UPS, undifferentiated pleomorphic sarcoma; LPS, liposarcoma; MFBS, myxoinflammatory fibroblastic sarcoma; HS, chondrosarcoma; MPNST, malignant peripheral nerve sheath tumor; SFT, solitary fibrous tumor; ALT, atypical lipomatous tumor; DFSP, dermatofibrosarcoma protuberans; WD-LPS, well-differentiated liposarcoma; (IR), intermediate-risk; (HR), high-risk; (LR), low-risk; LR, local relapse; RR, relapse in the regional lymph nodes; DR, distant relapse; DOD, died of disease; AWD, alive with disease; *, died of an unrelated cause/unknown.

## Data Availability

Data is contained within the manuscript. Additional information about the data may be provided on reasonable request.

## Abbreviations

The following abbreviations are used in this manuscript:

AJCC	American Joint Committee on Cancer
ALT	Atypical lipomatous tumor
ASA	American Society of Anesthesiologists
AWD	Alive with disease
CD	Clavien–Dindo classification system
CD34+	Cluster of differentiation 34-positive
CI	Confidence interval
CT	Chemotherapy
DFSP	Dermatofibrosarcoma protuberans
DM	Distant metastasis
DMFS	Distant metastasis-free survival
DOD	Died of disease
DR	Distant relapse
DSS	Disease-specific survival
ESTS	Extremity soft tissue sarcoma
FNCLCC	Fédération Nationale des Centres de Lutte Contre le Cancer
HR	Hazard ratio
HRG	High-risk group
HS	Chondrosarcoma
ILP	Isolated limb perfusion
IOLCC	Institute of Oncology Ljubljana Cancer Center
IQR	Interquartile range
LPS	Liposarcoma
LR	Local recurrence/local relapse
LRFS	Local recurrence-free survival
LRG	Low-risk group
MFS	Myxofibrosarcoma
MFBS	Myxoinflammatory fibroblastic sarcoma
MPNST	Malignant peripheral nerve sheath tumor
NA	Not applicable
OECI	Organisation of European Cancer Institutes
OR	Odds ratio
OS	Overall survival
Q1	First quartile
Q3	Third quartile
R0	Microscopically negative resection margin
R1	Microscopically positive resection margin
RR	Regional recurrence/regional lymph node relapse
RT	Radiotherapy
RT-CT	Radiotherapy and chemotherapy treatment
SFT	Solitary fibrous tumor
STS	Soft tissue sarcoma
UPS	Undifferentiated pleomorphic sarcoma
WD-LPS	Well-differentiated liposarcoma
WHO	World Health Organization
